# Non-human and human service efficiency of long-term care facilities in China

**DOI:** 10.3389/fpubh.2023.1066190

**Published:** 2023-03-02

**Authors:** Liangwen Zhang, Ying Han, Ya Fang

**Affiliations:** ^1^State Key Laboratory of Molecular Vaccinology and Molecular Diagnostics, School of Public Health, Xiamen University, Xiamen, China; ^2^School of Economics, Xiamen University, Xiamen, China

**Keywords:** data envelopment analysis, spatial Markov chain, service efficiency, long-term care facilities, non-human and human efficiency

## Abstract

**Introduction:**

Care services provided by long-term care facilities (LTCFs) are currently plagued by care resource shortages and insufficient utilization. The analysis on the temporal and spatial distribution of human resources and non-human resources in LTCFs, could provide a basis to optimize resource allocation and efficient use of limited resources.

**Methods:**

This study used data envelopment analysis to comprehensively evaluate the efficiency of human and non-human resources in different time spans and regions. The spatial Markov chain and spatial correlation were also applied to explore the heterogeneity of and correlation between the service efficiency of LTCFs in different regions and then analyzes the influencing factors of efficiency using Tobit regression model.

**Results:**

The quantitative changes in the service efficiency of LTCFs in various provinces showed a “W” shape in two periods, ranging from 0.8 to 1.6. The overall efficiency of LTCFs in different regions had a lower probability to achieve short-term cross-stage development. Non-human resource efficiency presented a “cluster” distribution mode, demonstrating a great probability to achieve cross-stage development, which might be due to the regional disparities of economic development and land resource. Tobit regression analysis results also showed that the comprehensive efficiency of LTCFs decreases by 0.210 for every square increase in construction space variation. However, human resource efficiency had a significant spatial polarization, making it difficult to develop area linkages. The reason for this might be the nursing staff have relatively stable regional characteristics, weakening the inter-provincial spatial connection. We also found that female workers, aged between 35 and 45 can positively affect the efficiency of LTCFs. Those staff stay focused and improve their skills, which might improve the efficiency of LTCFs. So improving technology and service quality changes by increasing female workers, aged between 35 and 45, and avoiding excessive construction space changes can enhance the growth of service quality and personnel stability of LTCFs.

**Conclusion:**

There is an urgent trade-off among staff quality improvement, resource reduction, construction excessive and substantial regional variation in efficiency. Therefore, strengthening policy support to encourage inter-regional initiatives, particularly highlighting the development of human resources interaction and common development is urgent.

## Introduction

The increasing life expectancy and declining fertility rate have led to a surge in the proportion of the older population, posing a severe global challenge of deep aging (1). Specifically, in China, the total number of people aged 60 years and above reached 264.02 million, accounting for 18.7% of the entire population in 2020, while the proportion is still rising, exceeding that of most EU countries (2, 3). A report in 2017 showed that there were about 40.63 million older adult people with disability in the country (4), and it is projected that 68 million older people in China will experience various degrees of disabilities in China by 2030, and approximately 18.6% of these people may require assistance for daily living activities (5). On the contrary, according to the first National Seminar on Intelligent Eldercare Strategy in 2012, the proportion of empty nesters will reach 54% by 2050 (6), which broke the traditional Chinese way to support families (7). As the family-oriented care function for older adults is constantly weakening, the demand for equitable and efficient long-term care facilities (LTCFs) will continue to increase (8). The Chinese government established 163,800 LTCFs with 3.928 million beds by the end of January 2019 (9). Total beds account for 3% of the country's older population. However, developed countries can reach 5–7%, namely, the number of beds required should be at least 12.45 million (10). In addition, only 300,000 registered long-term care workers cannot meet the care needs of 40 million older people with disabilities (8, 11). The increasing demand for human and non-human care services makes the service supply of LTCFs obviously insufficient (12).

There also existed weak quality regulation and insufficient utilization of efficiency of LTCFs, resulting in the current situation of care resource waste and imbalance development of LTCFs. For example, in the “Research Report on the Development of China's LTCFs,” it was highlighted that approximately 257 LTCFs in 12 interviewed cities, including Tianjin, Harbin, Jinan, and Wuhan; among those, about 32.5% were in the deficit of income (13). In addition, the deaths from the 2019 novel coronavirus disease (COVID-19) are heavily skewed toward older individuals (14). A report referred that the epidemics of COVID-19 has caused a 20% loss of revenue and a 30% increase in operating costs for long-term care facilities (LTCFs) (11). Furthermore, surveys showed that the phenomenon of “one bed is hard to find” in public LTCFs and “more than half of the beds are vacant” in private LTCFs is becoming increasingly obvious (15, 16). Even though 63% of older people who need eldercare are waiting for LTCFs, there still exist 54% of the beds in private institutions are vacant (17). Some of the LTCFs might be too expensive for older adults to afford. The other LTCFs have lower prices, but due to poor service quality, insufficient resources, and facilities, vacancy is also apparent (18). These presented the imbalance in the utilization of care resources by different types of LTCFs, and most institutions have far lower efficiency than their projected level, thus they cannot meet the needs of older adults (19). Therefore, how to further improve the service quality of LTCFs, reasonably allocate human and non-human service resources, increase their input-output benefits, improve service efficiency, and realize the sustainable development of LTCFs has become a concern and urgent problem to be solved by government departments.

With the deepening of the research on the service quality of LTCFs, foreign scholars began to pay more attention to the problem of inputs and outputs of LTCFs at the same time. A sample survey was conducted in nursing homes in certain areas of Finland, Italy, Japan, and Norway, which investigated the relative efficiency and influencing factors, based on explorations on the application of the data envelopment analysis (DEA) method and regression model (20–23). DEA was defined as an advantageous non-parametric technique for evaluating performance in terms of relative efficiency and can deal with multiple inputs and outputs (24). Also, it does not need to set an artificial weight coefficient (25). In addition, the DEA has no direct relationship with the dimensions of input and output indicators, and when it comes to service quality and cost-effectiveness, it does not need to calculate the health status and standard cost of service individuals, which applies to various situations with data limitation (26).

Although most of the studies in developed countries on the service efficiency of LTCFs are relatively complete, their research approaches and results may not apply to China due to different levels of development. There still exist amounts of shortcomings in the studies related to the efficiency of China's LTCFs. For example, scholars focused only on theoretical study for institutional planning of LTCFs, including the exploration of institution building and service standards, service contents, and service personnel. Some conducted a theoretical and analytical study for institutional planning of LTCFs in a certain province or city (27), being short of quantitative studies, which cannot provide a scientific basis for promoting the overall healthy development of nursing homes in China from a macro perspective (28). In addition, the existing studies are often limited to use the cross-sectional data to apply the single stage (29) and DEA Tobit two-stage models in conducting regional static research (30), less considering the application of longitudinal data from time and regional dynamic analysis of service efficiency changes and its influencing factors exploration.

The analysis of the dynamic spatial layout of medical facilities in China and abroad is mainly based on the impact of the service needs of patients on the spatial distribution of institutions. The accessibility of healthcare and hospitals and surrounding environmental factors related to the needs of the patients are mainly studied by means of network analysis, geographic information system (GIS) (31), spatial sentence (32), social network analysis (SNA) (33), enhanced two-step floating catchment area (E2SFCA) (34), multivariable regression methods (35), ordinary least squares (OLS) (36), spatial autoregressive (SAR), and geographically weighted regression (GWR) (37) in the community prevention and healthcare facilities. Christoph Pross specified a Bayesian, geoadditive, Stochastic Frontier Analysis (SFA) model to assess hospital performance along the dimensions of resources and quality of stroke care in German hospitals (38). Wu Anqi (39) explored the current situation of the spatial distribution of LTCFs and the accessibility of LTCFs in Lanzhou. Regional growth convergence has always been the main topic in the empirical study of efficient growth. Quah proposed to use kernel density estimation and Markov chain methods to study the evolution of regional growth distribution (40). The spatial Markov chain analysis method is used to test whether the transfer dynamics faced by a region are affected by the efficient level of neighboring regions (41). However, the conditional convergence test data without considering geographical features might inevitably result in serious deviation when evaluating the quantitative of the pension industry agglomeration (42). Thus, Rey proposed a new index based on the reconstruction of the spatial decomposition of the classical Gini coefficient (i.e., the Moran index), which is highly correlated with spatial autocorrelation and data distribution (43). Chinese provinces and cities have been maintaining a rapid development of LTCFs, but there is also a lack of coordination between regions. It is an important empirical problem to measure the growth or convergence of service efficiency of LTCFs in different regions combined with spatial Markov chain analysis and the Moran index.

Spatial distribution and spatial spillover effects are the focus of the studies on the spatial characteristics of LTCFs (44). However, the analysis results of service efficiency and resource allocation of LTCFs are not expressed in space from the supply side, and there is a lack of dynamic analysis of the service efficiency of LTCFs across time, space, and region. In addition, the existing studies used cross-sectional data, and the LTCFs were restricted regionally, which did not have the representativeness of the overall characteristic distribution, and the research conclusions lacked practicality and extension. Furthermore, the current LTCFs efficiency literature has rarely taken into account the importance of geographic clusters (30). Neglecting the efficiency of LTCFs in the same or neighboring regions might induce systematic biases to inefficiency estimated effects of their determinants if efficiency varies not only between providers but also across regions. However, current studies only focus on regional differences, spatial distribution, and spatio-temporal dynamic evolution in hospitals and the healthcare field (45). Taking the socioeconomic and technological attributes of LTCFs as the basis, it is necessary to further study whether the inputs and outputs of LTCFs as a production activity in different regions are reasonable, as well as their spatial connections, and then to explore their influence mechanism and effective promotion path.

Therefore, combined with the Pareto optimal theory, we selected the input-output indicators for the efficiency evaluation of LTCFs from the aspects of human and non-human resources and applied the super efficiency model in data envelopment analysis to measure, rank, and classify the efficiency of 31 provinces and regions in China. Based on this, spatial autocorrelation, Markov chain, and spatial Markov chain were used to explore the regional differences and dynamic evolution of the service efficiency of LTCFs in the context of aging by considering areas and time heterogeneity, and then to analyze the influencing factors of LTCFs' efficiency by panel Tobit regression. The goal of this study is to provide a more complete understanding of the dynamic spatial distribution changes and the influencing factors of the decrease in the human and non-human resource efficiency of LTCFs at the national level. This is done in order to reveal the spatial changes and connections of LTCF's efficiency as influenced by different factors and explore the path to achieve higher efficiency. In turn, it provides a basic reference for the government to optimize the allocation of eldercare service resources and the spatial pattern of eldercare services and then to improve the service quality.

## Methods

### Data sources

Data were obtained from the China Civil Affairs Statistical Yearbook, a national survey compiled by the Statistical Annual Report of the Social Service Industry, and the reports of relevant departments, which are related to social services in each year among all LTCFs in the 31 provinces of China from 2013 to 2020. The data content includes the input and output indicators of LTCFs, where the input indicators include human resources indicators and non-human resources indicators.

### Variable selection

There is no consensus conclusion on the standard of input-output variables for the evaluation of the efficiency of LTCFs (46, 47). Based on the theory of production factors in economics, the principles of representativeness, independence, and operability should be included in the evaluation index selection. To be more specific, input factors can be divided into capital, labor, and material inputs, and output factors include economic and social benefits (48).

Considering the operability of decisions, input indicators should include the items that decision makers can control over and modify (49). As LTCFs were labor-intensive industries, fixed assets and institutional staff were commonly used separately as capital input human input index (50, 51). The actual number of beds could be easily controlled by managers, which belongs to material input. A number of studies also considered the number of beds, the number of institutions, and the original price of fixed assets as indicators of material resources (27, 52, 53). In addition, as the pension industry functions as a labor-intensive industry, there is less possibility for the capital of the labor substitution. Hence, human capital is an appropriate input index for efficiency comparison including the number of social workers and the number of employees, which are the main care resources in LTCFs (54). Therefore, the input indicators established in this study include the number of institutions, the number of employees at the end of the year, the original price of fixed assets, the number of social workers, and the number of beds at the end of the year (Table 1).

**Table 1:** The overall efficiency evaluation of LTCFs in China in 2020.

**DMU**	**TE**	**PTE**	**SE**	**RTS**	**SBM**	**Rank**
Beijing	1	1	1	CRS	2.075259	3
Tianjin	1	1	1	CRS	1.113012	9
Hebei	0.923478	0.941243	0.981125	CRS	0.660007	27
Shanxi	0.663327	0.804785	0.824229	CRS	0.537396	30
Inner Mongolia	0.88907	0.905911	0.98141	CRS	0.767648	23
Liaoning	1	1	1	CRS	1.065222	13
Jilin	1	1	1	CRS	1.151649	7
Heilongjiang	0.806433	0.913878	0.88243	DRS	0.742034	24
Shanghai	1	1	1	CRS	2.765687	2
Jiangsu	0.863247	1	0.863247	DRS	1	17
Zhejiang	1	1	1	CRS	1.093885	10
Anhui	0.988325	0.989857	0.998452	CRS	0.928381	22
Fujian	0.920666	0.921996	0.998557	CRS	0.697082	26
Jiangxi	1	1	1	CRS	1.801637	4
Shandong	0.910557	1	0.910557	DRS	1	18
Henan	1	1	1	CRS	1	19
Hubei	1	1	1	CRS	1.148148	8
Hunan	1	1	1	CRS	1.081579	11
Guangdong	0.939754	1	0.939754	DRS	1	20
Guangxi	0.848276	0.849864	0.998132	CRS	0.524982	31
Hainan	1	1	1	CRS	1.552696	5
Chongqing	0.862676	1	0.862676	DRS	1.010352	16
Sichuan	1	1	1	CRS	1	21
Guizhou	0.736317	0.820939	0.896921	CRS	0.605067	28
Yunnan	0.80127	0.80203	0.999052	CRS	0.59572	29
Tibet	1	1	1	CRS	4.366281	1
Shaanxi	0.980765	1	0.980765	DRS	1.014799	14
Gansu	0.986527	0.986827	0.999696	CRS	0.717468	25
Qinghai	1	1	1	CRS	1.162113	6
Ningxia	1	1	1	CRS	1.066009	12
Xinjiang	1	1	1	CRS	1.014693	15

TE, technical efficiency; PTE, pure technical efficiency; SE, scale efficiency. Achieved optimal efficiency if the value is 1, while the efficiency is low if it is < 1. The higher the super-SBM efficiency index, the higher the efficiency.

Output indicators are considered to be the most important factors in evaluating the quality and quantity of long-term care services (55). However, most studies only considered the output of operating revenue and profit, and the number of patients and beds, rarely considering the importance of service quality in evaluating the efficiency of LTCFs (56, 57). The quality of service directly affects the final effect of eldercare and the development prospects of LTCFs. For example, the health condition of older people is very important for the improvement and development of LTCFs, and it has a direct impact on the final performance evaluation results of the service such as the number of rehabilitation and medical outpatients. The quality of service is based on the fall rates of older adults, health conditions, the rate of complaint handling, and the annual incidence of major accidents (58). Furthermore, the number of older people with different care needs can reflect their social effects, and thus older population was classified into disabled, partially disabled, and completely independent in LTCFs, which were measured by the Barthel index including six basic activities of daily living, namely, eating, toileting, bathing, dressing, getting in and out of bed, and mobility (30). Therefore, the output indexes should include operating income, the number of disabled, the number of partially disabled, the number of independents in residential cares at the end of the year, and the number of rehabilitation and medical outpatients (Supplementary Table S1).

According to the summary statistics of input-output variables presented, the mean number of institutions reached the highest in 2014. All the input indexes have a significant correlation with output indexes, which indicates that the index selected is reasonable (Supplementary Tables S2, S3).

### Data envelopment analysis

Data envelopment analysis is a linear programming technique proposed by Charnes et al. to deal with evaluation problems containing multiple input and output indicators (59), which are categorized into input- and output-oriented. Considering the input controllability of the pension industry and the limited eldercare resources in China, it is of great practical significance to increase the output with the given input to make more reasonable use of resources. Therefore, input-oriented variable scale DEA models, i.e., the generalized Charnes-Cooper-Rhodes (CCR) and Banker-Charnes Cooper (BCC) models, were used to analyze the comprehensive efficiency of LTCFs. The provincial domain is set as a decision unit in combination with the actual, i.e., a total of 31 decision units, which are presented by DMU (*i* = 1, 2, ……, *n*) (25). The calculation was adopted by the MaxDEA software.

#### CCR and BCC models

Data envelopment analysis is a frontier analysis technology based on linear programming. It uses management operations research to construct the production frontier (optimal envelope), then maps the data of the evaluated unit into space, and calculates the relative efficiency value according to the mapped position. It calculates the relative efficiency value according to the mapped position: the relative efficiency value falling on the most envelope line is 1 to achieve the optimal efficiency; if it falls at other positions, the relative efficiency value is calculated according to the position, and the efficiency value is < 1. Therefore, the efficiency value interval obtained by using traditional DEA is (0,1) (60). The traditional DEA model mainly included CCR model l (61) based on constant returns to scale and the BCC model with variable returns to scale (62). The CCR model calculates for technical efficiency (TE), while the BCC model calculates for pure technical efficiency (PTE). The TE refers to the extent to which a DMU can produce the maximum output from its chosen combination of inputs, PTE refers to the production efficiency affected by management and technology. SE refers to the production efficiency affected by constructions or scale factors. The relationship formula of these three values is *TE* = *PTE*×*SE*.

#### Slack-based model (SBM) model

The additive model (AM) or SBM is based on input and output slacks. In this study, a non-oriented and non-radial model known as the SBM-DEA model has been used (63). This model breaks through the limitation that the maximum efficiency value of the traditional DEA can only be 1 (64). It takes the optimal envelope curve as the benchmark, gives the relative mapping position of each evaluated unit, sorts the optimal units with the original efficiency value of 1, and can reflect the specific relative efficiency value of the optimal efficiency unit. Therefore, the value of super efficiency becomes (0, ∞). The input-oriented DEA model was used to compute TE scores of nursing care that can be expressed by the following formula:


$$\{\begin{array}{l} \max\left[\theta -\varepsilon \left(e^{T}s^{-}+{\hat{e}}^{T}s^{+}\right)\right]\\ \sum_{j=1}^{n}x_{j}\lambda_{j}{\text{+s}}^{-}=\theta x_{0}\\ \sum_{j=1}^{n}y_{j}\lambda_{j}-s^{\text{+}}\text{=}y_{0}\\ \lambda_{j}\geq 0,j=1,...,n,s^{+}\geq 0;s^{-}\geq 0 \end{array}$$

In the case of θ = 1, *S*^−^ = 0, *S*^+^ = 0, the nursing home is fully efficient, whereas θ < 1 means that a nursing home is inefficient. The BCC model adds constraint conditions based on the CCR model:


$$\begin{array}{l} \overset{\text{n}}{\underset{j=1}{\sum }}\lambda_{j}=1 \end{array}$$

At this time, it means that the return on the scale of DMU remains unchanged and reaches the maximum output scale (65). In addition, when $\sum_{j=1}^{n}\lambda_{j}=1<1$, it means that returns to scale are increasing. If the input to DMU is appropriately increased based on the original input, the output will be increased by a higher proportion. When $\sum_{j=1}^{n}\lambda_{j}=1>1$, it means diminishing returns to scale, and the increasing input does not lead to a higher proportion of output (66).

#### Malmquist model

Productivity measures changes in a production unit's efficiency in transforming inputs into outputs from time t to time *t*+1 (67). Total factor productivity changes (TFPC) can be decomposed into technical efficiency changes (TEC) and technological changes (TC). TEC can also be decomposed into pure technical efficiency changes (PTEC) and scale efficiency changes (SEC). The formula is as follows:


$$\begin{array}{l} \text{TFPC}=\text{TEC}\times \text{TC}=\left(\text{PTEC}\times \text{SEC}\right)\times \text{TC} \end{array}$$

### Spatial statistical analysis

#### Time heterogeneity test

The method of testing time homogeneity was proposed by Anderson and Goodman (68). The whole sample is divided into *t*-period, and the Pearson χ^2^ and likelihood ratio are used to test whether the transfer matrix estimated from each *t*-sub sample is significantly different from the matrix estimated from the whole sample.

#### Spatial Markov chain

Markov chain is a kind of Markov process whose time and state are discrete. It can be used to study the random transfer of economic phenomena without the interference of external factors. The specific formula is as follows:


(1) $$\begin{array}{l} M_{ij}=n_{ij}/n_{i} \end{array}$$

In Equation (1), *n* represents the sum of the number of spatial units from type *i* tourism efficiency at time *t* to type *j* tourism efficiency at time *t*+*n*; *n* represents the sum of the number of spatial units of type *i* at all times during the study period, and *M* represents the probability value that the tourism efficiency of spatial units of type *i* at time *t* changes to type *j* at time *t*+*n*.

Through the spatial Markov chain, based on the adjacent spatial weight matrix and taking the spatial lag of the research unit in the initial year as the premise, the traditional Markov transition probability matrix is decomposed into *k* conditional transition probability matrices of *k*×*k*. The specific formula is as follows:


(2) $$\begin{array}{l} \text{Lag}=Y_{i}W_{ij} \end{array}$$

In Equation (2), *y* is the attribute value of the spatial unit; *W* is the element of row *i* and column *j* of the spatial adjacency weight matrix *W*.

#### Spatial autocorrelation analysis

##### Global spatial autocorrelation

Moran index can describe the spatial agglomeration degree of tourism efficiency of each province. When the spatial weight matrix is constructed according to the adjacency principle, the value range of the global Moran index is [-1,1]. When its index is not equal to 0, it indicates that there is a spatial positive autocorrelation or negative correlation between the service efficiency of provincial LTCFs.


(3) $${\text{Moran}}^{\prime }\text{s I=}\frac{\sum_{\mathrm{i=1}}^{n}\sum_{\mathrm{j=1}}^{m}W_{\mathrm{ij}}(X_{i}-\bar{X})(X_{j}-\bar{X})}{S^{2}\sum_{\mathrm{i=1}}^{n}\sum_{\mathrm{j=1}}^{m}W_{\mathrm{ij}}}\in [-1,1]$$

In Equation (3), *X*_*i*_ is the observation value, *W*_*ij*_is the spatial weight matrix, and the spatial autocorrelation matrix selects the adjacency matrix.

##### Local spatial autocorrelation

To measure the local correlation property of each provincial unit, the local spatial autocorrelation coefficient is introduced to further identify the spatial correlation pattern of local areas (69).


(4) $$\begin{array}{l} LISA_{i}=Z_{i}\overset{n}{\underset{j=1}{\sum }}WZ_{j} \end{array}$$

In Equation (3), *LISA*_*i*_ is the local spatial autocorrelation coefficient; *Z*_*i*_ and *Z*_*j*_ are the standardized values of service efficiency of LTCFs of provinces. When the value of *LISA*_*i*_ is positive, it means that the similarity value of local spatial units tends to be spatially concentrated, and when it is negative, it means that local spatial units tend to be dispersed.

### Tobit regression model

The Tobit model is suitable for the research of the regression equation with a limited value of the dependent variable and is conducive to accurately measuring the influence of the independent variable on the dependent variable (70). As the operating efficiency of LTCFs measured by the DEA model is between 0 and 1, the least square method (OLS) may cause deviation in the results, so the panel Tobit model with limited dependent variables is adopted for analysis, which can better solve the regression problem with limited dependent variables (71). In addition, the efficiency of LTCFs is affected by not only the five input factor variables but also other exogenous variables (72). According to the availability of relevant literature and data, it mainly discusses three aspects, namely, service subjects, human resources, and non-human resources. Among them, service subjects include the type of object of service (self-paid, extreme poverty, and entitled groups) and the total number of subjects in LTCFs. Human resources include the influence of gender, educational background (junior college, college graduate, and above), age (35 years old or below, 36–45, 46–55, or above 56 years old), the number of assistant social workers, the number of volunteer service personnel, volunteer service time, and other factors on the efficiency. Non-human resources include construction space. To avoid the possible heteroscedasticity of the data and improve the convergence rate of the model, the values of the total number of variables were normalized before regression analysis (73). Taking comprehensive efficiency as a dependent variable, a panel data Tobit regression analysis was carried out to find out the factors affecting operation efficiency (74). The model is as follows:


$$Y={\{}_{0,Y^{*}\leq 0}^{Y^{*}=\alpha +\beta X+\mu ,\;Y^{*}>0}\;$$

where Y^*^ is the restricted dependent variable, *X* is the vector of independent variables, α is the vector of intercept terms, β is the vector of regression parameters, μ is the random error term, and μ~*N*(0, σ^2^).


$$\begin{array}{l} EFF_{it}=\alpha_{i}+\beta_{1}X_{1}+\beta_{2}X_{2}+\beta_{3}X_{3}+\beta_{4}X_{4}+\beta_{5}X_{5}+\beta_{6}X_{6}\\ \;+\beta_{7}X_{7}\sim \beta_{15}X_{15}+\varepsilon_{it}\; \end{array}$$

All statistical analyses were performed using MATLAB, Stata 16.0, DEA solver 5.0, and GeoDa software.

## Results

### The evolutionary trends of the efficiency of LTCFs from 2013 to 2020

**Figure 1:**
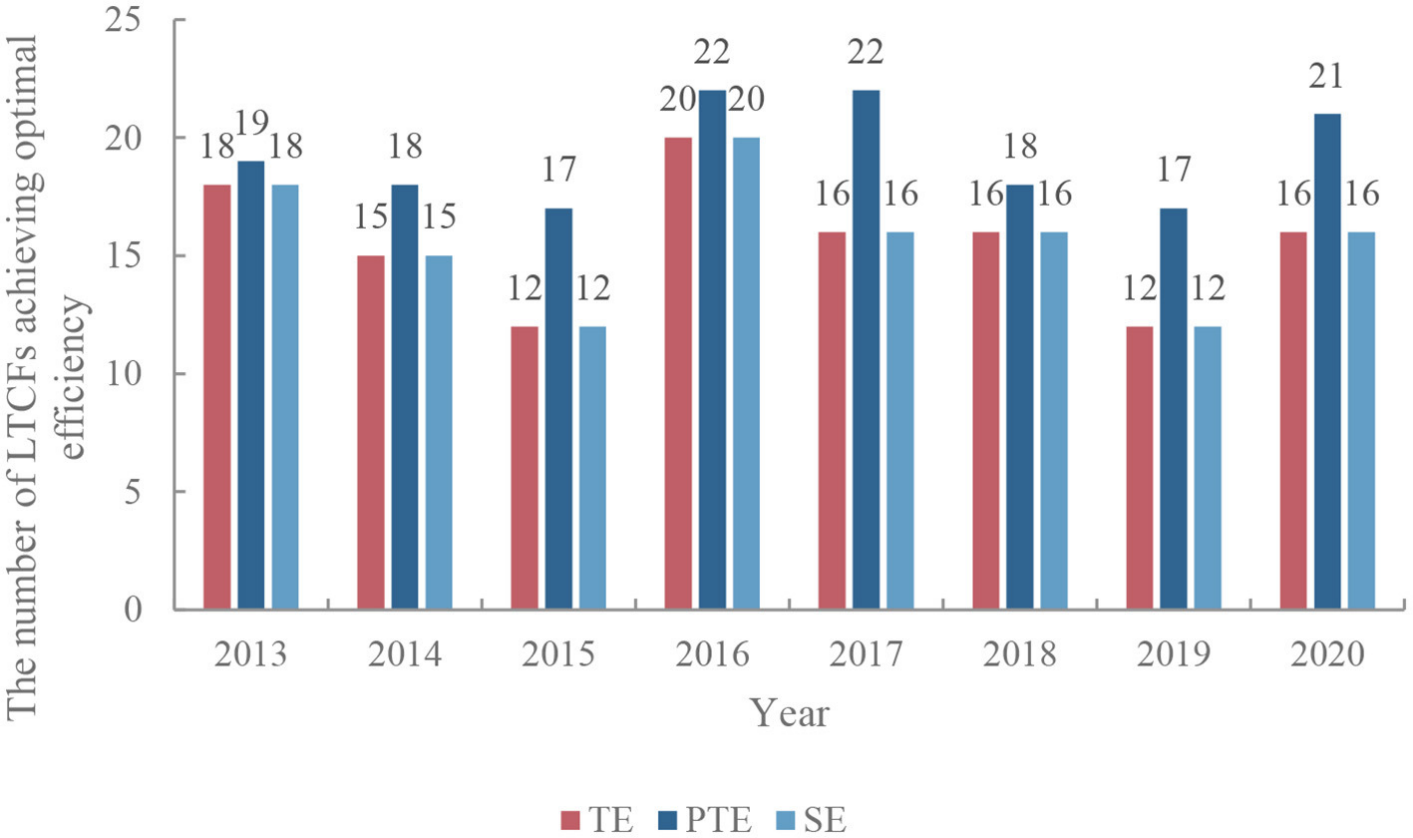
The number of LTCFs with optimized service efficiency in China (2013–2020). TE, technical efficiency; PTE, pure technical efficiency; SE, scale efficiency.

The results of the DEA-BCC-CCR method showed that the number of LTCFs that achieved the optimal efficiency in TE, PTE, and SE presented a composite W-shape trend of change from 2013 to 2020. The number of institutions that have reached the optimal efficiency values in TE, PTE, and SE reached the lowest level in 2015 and 2019 and reached the highest level in 2016 (Figure 1).

### The efficiency evaluation of LTCFs in China in 2020

In 2020, the overall efficiency value of LTCFs in 16 provinces (51.61%) was 1, which was in constant returns to scale, and the LTCFs in 6 provinces (19.35%) were in the stage of decreasing returns to scale. The super-SBM efficiency of LTCFs in Tibet ranked the highest, followed by Shanghai. The ranking of LTCFs in Guangxi Province is the lowest, followed by Shanxi Province (Table 1).

**Figure 2:**
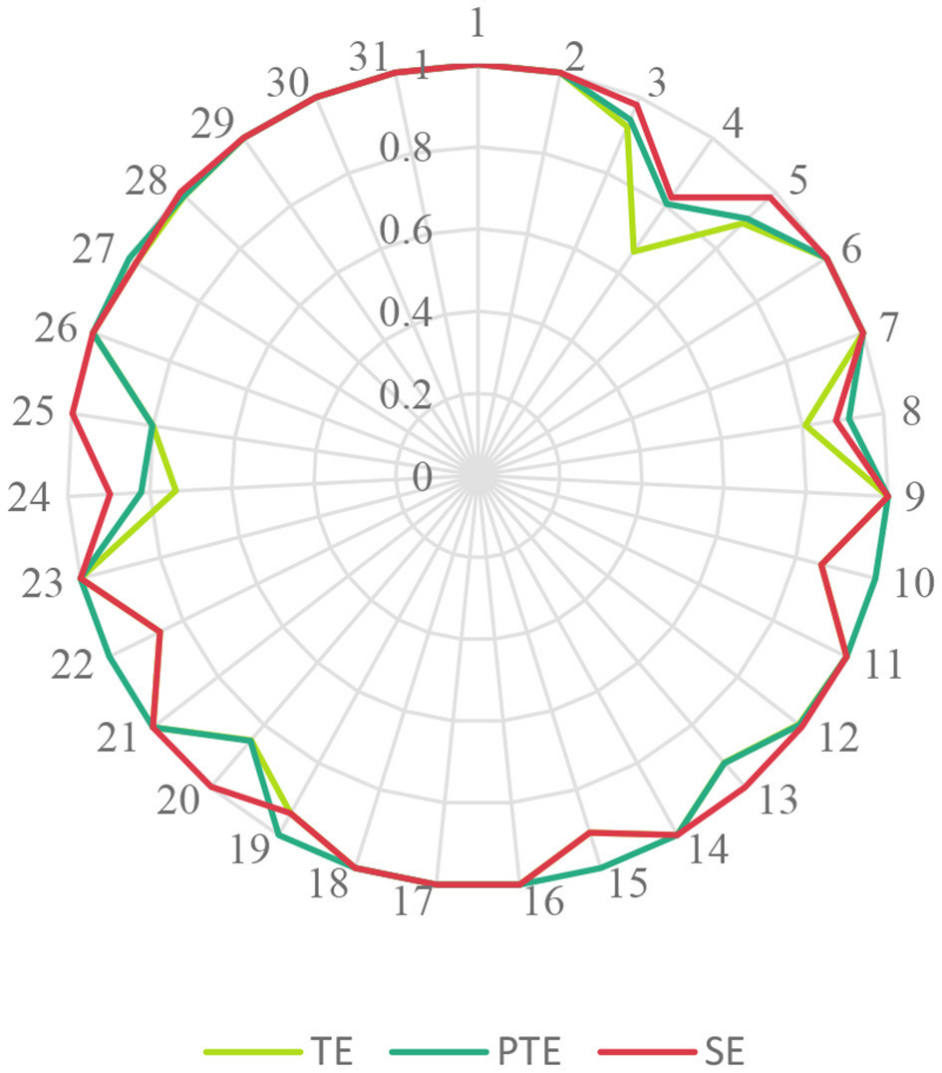
Distribution of TE, PTE, and SE of LTCFs in all provinces in 2020. TE, technical efficiency; PTE, pure technical efficiency; SE, scale efficiency.

Combined with the radar map of the service efficiency distribution of LTCFs in all provinces in 2020 (Figure 2), it showed that the efficiency of various LTCFs varies significantly among all the units.

### Malmquist total factor production and its decomposition from 2013 to 2020

Malmquist intertemporal analysis demonstrated that the decrease in total factor productivity in LTCFs in 24 provinces is mainly caused by the decrease in technology. At the same time, the decrease in TE of LTCFs in six provinces is caused by a decrease in SE, in another four provinces by a decrease in PTE, and in three provinces by a decrease in both PTE and SE (Table 2; Supplementary Table S4).

**Table 2:** Intertemporal analysis on the overall efficiency of LTCFs in 31 provinces (2013–2020).

**DMU**	**TEC**	**TC**	**PTEC**	**SEC**	**TFPC**
Beijing	1	1	1	1	1
Tianjin	1	0.974	1	1	0.974
Hebei	1.015	0.963	1.012	1.003	0.977
Shanxi	1.007	0.945	1.011	0.996	0.952
Inner Mongolia	1.03	0.982	1.03	1	1.011
Liaoning	1.043	0.936	1.036	1.007	0.976
Jilin	1	0.977	1	1	0.977
Heilongjiang	0.985	0.948	0.987	0.998	0.934
Shanghai	1	1.036	1	1	1.036
Jiangsu	0.991	0.973	1	0.991	0.964
Zhejiang	1	0.981	1	1	0.981
Anhui	1.004	0.935	1.004	1	0.939
Fujian	1.083	0.942	1.081	1.002	1.02
Jiangxi	1	0.973	1	1	0.973
Shandong	0.989	0.908	1	0.989	0.898
Henan	1	0.991	1	1	0.991
Hubei	1	0.874	1	1	0.874
Hunan	1.006	0.959	1.003	1.003	0.965
Guangdong	0.992	0.974	1	0.992	0.966
Guangxi	0.989	1.002	0.986	1.003	0.99
Hainan	1	1.003	1	1	1.003
Chongqing	1.01	0.955	1.01	1.001	0.964
Sichuan	1	0.895	1	1	0.895
Guizhou	0.97	0.869	0.972	0.998	0.843
Yunnan	0.972	0.953	0.974	0.998	0.927
Tibet	1.008	1.140	1	1.008	1.149
Shaanxi	1	0.939	1	1	0.939
Gansu	1.034	0.966	1.031	1.002	0.999
Qinghai	1	0.903	1	1	0.903
Ningxia	1	0.873	1	1	0.873
Xinjiang	1.028	0.922	1.023	1.004	0.948

TFPC, total factor productivity changes; PTEC, pure technical efficiency changes; SEC, scale efficiency changes; TC, technological changes; TEC, technical efficiency changes.

### Dynamic evolution analysis of service efficiency distribution among LTCFs

The test statistics used are Pearson χ^2^ statistics (*q*) and likelihood ratio statistics (*LR*) for the time heterogeneity test. The results show that both statistics reject the assumption of smooth transition probability at a significance level of 0.01. Therefore, the following will be divided into two periods, i.e., 2013–2017 and 2018–2020 (Supplementary Table S5).

According to the quantile rule [within 25% is low level (L), 26–50% is medium and low level (ML), 51–75% is medium and high level (MH), and >75% is high level (H)], the service efficiency of provincial LTCFs is divided into four categories, and the changes in transition probability are compared between two periods, i.e., 2013–2017 and 2018–2020. The results of the traditional Markov transition probability matrix showed that the diagonal probability of the super-SBM value is significantly higher than that of other positions, indicating that in the medium or short term, the efficiency development of LTCFs in China is relatively stable. This implies that it is difficult for provinces to realize the transition by relying on their own development without external causes. However, adjacent level types reached a large transfer probability, which might be the reason that industrial accumulation is the main development process in the current situation. Comparing the efficiency of human resources with that of non-human resources in LTCFs, the diagonal and other positions of human resources were more different in the 2013–2017 period with worse liquidity. However, in the 2018–2020 period, the liquidity was significantly higher than that of non-human resources, and there was a greater probability of cross-level development (Table 3).

**Table 3:** Dynamic evolution analysis of service efficiency distribution of LTCFs in two time periods (2013–2017 and 2018–2020) in China.

**Year**	**Level**	**Super-SBM**				**Human resource efficiency**				**Non-human resource efficiency**			
		**L**	**ML**	**MH**	**H**	**L**	**ML**	**MH**	**H**	**L**	**ML**	**MH**	**H**
2013–2017	L	0.563	0.188	0.188	0.063	0.594	0.375	0.000	0.031	0.563	0.219	0.063	0.156
	ML	0.162	0.676	0.081	0.081	0.212	0.424	0.182	0.182	0.163	0.651	0.070	0.116
	MH	0.231	0.154	0.308	0.308	0.067	0.100	0.667	0.167	0.167	0.278	0.278	0.278
	H	0.034	0.034	0.276	0.655	0.103	0.103	0.103	0.690	0.065	0.097	0.258	0.581
2018–2020	L	0.688	0.250	0.063	0.000	0.667	0.200	0.133	0.000	0.733	0.267	0.000	0.000
	ML	0.053	0.632	0.211	0.105	0.176	0.412	0.294	0.118	0.083	0.583	0.167	0.167
	MH	0.167	0.083	0.583	0.167	0.000	0.214	0.643	0.143	0.111	0.222	0.222	0.444
	H	0.000	0.133	0.067	0.800	0.188	0.063	0.063	0.688	0.071	0.214	0.071	0.643

L, low level; ML, medium and low level; MH, medium and high level; H, high level.

After adding the spatial correlation matrix, the results of the service efficiency of national LTCFs also showed a stable transition probability, including the human resource efficiency and non-human resource efficiency of LTCFs. For example, when the overall efficiency is in an L, ML, or H adjacent area, the diagonal transition probability is higher than that of other locations. Only when the adjacent area is MH, provinces with MH have a high probability of transferring to the adjacent horizontal section, ML or H. In human resource efficiency, when the adjacent area is L, the provinces that are L and MH have a higher probability of transferring to ML and L, respectively; when the adjacent area is ML, the provinces that are ML will have a greater probability of transferring to L; when the adjacent area is H, the provinces with L have a greater probability of transferring to ML; and when the adjacent area is MH, the diagonal transfer probability is higher than that of other locations. In terms of non-human resource efficiency, when the adjacent area is ML, the provinces with MH are more likely to transfer to ML; when the adjacent area is MH, the provinces that are MH themselves are more likely to transfer to L and H; and when the adjacent area is H, the provinces with MH themselves have a greater probability of transferring to H. The diagonal probability values of other adjacent space transfer matrices are much greater than those of other positions, which reflects the convergence of mechanism efficiency space. This is consistent with the results of spatial autocorrelation, which showed that LTCFs continue to form industrial clusters in their own regions in the process of development with high homogeneity (Table 4).

**Table 4:** Spatial Markov chains.

		**Total**				**Human resource efficiency**				**Non-human resource efficiency**			
**Level**	**T** *_*i*_* **/T**_i+1_	**L**	**ML**	**MH**	**H**	**L**	**ML**	**MH**	**H**	**L**	**ML**	**MH**	**H**
L	L	0.500	0.167	0.167	0.167	0.200	0.600	0.000	0.200	0.429	0.143	0.000	0.429
	ML	0.000	0.833	0.000	0.167	0.000	0.833	0.167	0.000	0.000	1.000	0.000	0.000
	MH	0.143	0.000	0.714	0.143	0.667	0.000	0.333	0.000	0.000	0.000	1.000	0.000
	H	0.000	0.000	0.333	0.667	0.333	0.000	0.000	0.667	0.100	0.000	0.300	0.600
ML	L	0.577	0.192	0.192	0.038	0.591	0.318	0.045	0.045	0.640	0.200	0.080	0.080
	ML	0.077	0.692	0.115	0.115	0.467	0.333	0.133	0.067	0.106	0.574	0.149	0.170
	MH	0.250	0.125	0.375	0.250	0.071	0.143	0.500	0.286	0.125	0.417	0.250	0.208
	H	0.077	0.077	0.269	0.577	0.200	0.100	0.000	0.700	0.067	0.167	0.200	0.567
MH	L	0.769	0.154	0.077	0.000	0.769	0.231	0.000	0.000	0.800	0.100	0.000	0.100
	ML	0.235	0.471	0.176	0.118	0.200	0.533	0.133	0.133	0.200	0.600	0.000	0.200
	MH	0.167	0.333	0.167	0.333	0.000	0.115	0.692	0.192	0.500	0.000	0.000	0.500
	H	0.000	0.222	0.111	0.667	0.061	0.091	0.182	0.667	0.000	0.500	0.000	0.500
H	L	0.556	0.222	0.222	0.000	0.429	0.500	0.071	0.000	0.500	0.500	0.000	0.000
	ML	0.250	0.688	0.063	0.000	0.267	0.333	0.267	0.133	0.375	0.500	0.125	0.000
	MH	0.143	0.286	0.286	0.286	0.143	0.357	0.357	0.143	0.000	0.000	0.250	0.750
	H	0.000	0.000	0.200	0.800	0.222	0.000	0.111	0.667	0.000	0.182	0.182	0.636

L, low level; ML, medium and low level; MH, medium and high level; H, high level.

### Spatial correlation analysis of overall efficiency and human and non-human resource efficiency of LTCFs

To explore the interactive effect of the development of LTCFs among provinces, Moran's I was used to reflect the spatial relationship among regions. According to Table 5, the non-human resource efficiency in different provinces had a weak spatial correlation in 2013–2015; the human resource efficiency had a weak spatial correlation only in 2017; and the overall efficiency of LTCFs failed to pass the test in 2013–2020. As for non-human resource efficiency, in the early stage of development, the LTCFs were not developed in all regions, and there was a certain linkage between the resource efficiency of various provinces. However, with the continuous development and integration of the local pension industry, provinces began to accumulate pension resources within their own province, causing the inter-provincial spatial connection to weaken. As for human resource efficiency, there is no spatial correlation between provinces. There was a negative spatial relationship that passed the 0.1 test in 2017, but Moran's I in the other years failed to pass the test. There existed a negative exponential phenomenon, which resulted in an obvious polarization phenomenon and failed to form area linkage to develop efficiency.

**Table 5:** The Moran's I of human resources and non-human resources in LTCFs.

**Year**	**Total efficiency**		**Human resource efficiency**		**Non-human resource efficiency**	
	**Moran's I**	**P**	**Moran's I**	***P***	**Moran's I**	**P**
2013	−0.123	0.167	−0.081	0.345	−0.141	0.099
2014	0.024	0.252	−0.059	0.471	0.081	0.079
2015	−0.162	0.108	−0.083	0.306	−0.179	0.072
2016	−0.046	0.498	−0.097	0.147	0.016	0.269
2017	−0.019	0.184	−0.006	0.074	−0.031	0.477
2018	0.031	0.239	0.110	0.113	0.016	0.202
2019	−0.024	0.384	−0.019	0.406	−0.018	0.369
2020	−0.040	0.449	−0.079	0.374	−0.012	0.314

### Analysis of local spatial autocorrelation aggregation of service efficiency of LTCFs in various provinces from 2013 to 2020

Supplementary Figures S1A–H shows that during the period 2013–2020, when the test level is at *p* ≤ 0.05, there is a significant positive spatial correlation (+ +) between one province and its adjacent provinces or regions, which can be identified as diffusion effect that shows a relatively rapid development model, and there is a significant positive spatial correlation (– –) between eight provinces and their neighboring provinces or regions. The efficiency growth rate of these provinces is relatively slow, showing a typical lagging development model; there is a significant negative spatial correlation (+ –) between one city and its adjacent cities, which can be identified as the polarization effect or reflux effect; and there is a significant negative spatial correlation (– +) between four provinces and their neighboring provinces, which can be identified as the centrifugal effect.

### The empirical analysis of influencing factors of LTCFs efficiency

Based on the Tobit model constructed in the previous section, the results showed that female workers aged between 35 and 45 years can positively affect the efficiency of LTCFs at a significant level of 1% and 5%. In addition, the type of service subjects has a positive impact on the efficiency of LTCFs. A possible reason for this is that those groups are generally in poorer health or able to receive more government subsidies from long-term care insurance, thus this income increases the efficiency of LTCFs.

However, the construction space of the LTCFs might negatively affect the efficiency of LTCFs at a 5% significant level. Table 6 shows that the comprehensive efficiency of LTCFs decreases by 0.210 for each square increase in construction space variation.

**Table 6:** Tobit regression analysis of the influential factors of LTCFs efficiency.

**Variables**	**Coef**.	**S. t**.	***T*–value**	***p–*value**	**95%** ***CI***	
Constant	0.9794	0.0456	21.4800	0.0000	0.8896	1.0692
**Support subjects**						
Entitled group	0.1308	0.0559	2.3400	**0.0200** ^*^	0.0207	0.2409
Extreme poverty group	0.2251	0.0662	3.4000	**0.0010** ^**^	0.0946	0.3556
Self-paid group	0.0986	0.0646	1.5300	0.1280	−0.0287	0.2259
Annual older people	0.0949	0.0566	1.6800	0.0950	−0.0167	0.2064
**Human resources**						
Women	0.3895	0.1276	3.0500	**0.0030** ^**^	0.1382	0.6409
Junior college	−0.1312	0.1017	−1.2900	0.1980	−0.3315	0.0692
College graduate and above	0.0895	0.0899	1.0000	0.3210	−0.0877	0.2666
Age d35 and below	−0.1508	0.1039	−1.4500	0.1480	−0.3556	0.0540
Aged between 35 to 45	−0.3176	0.1314	−2.4200	**0.0160** ^*^	−0.5764	−0.0588
Aged between 46 to 55	−0.0191	0.0878	−0.2200	0.8280	−0.1920	0.1538
Aged 56 and above	0.1321	0.0825	1.6000	0.1110	−0.0305	0.2947
Social workers	0.0321	0.0512	0.6300	0.5320	−0.0688	0.1330
The numbers of volunteers	0.0275	0.1244	0.2200	0.8250	−0.2176	0.2726
Volunteer service hours	0.1321	0.1423	0.9300	0.3540	−0.1482	0.4125
**Non-human resources**						
Construction space	−0.2101	0.0716	−2.9300	**0.0040** ^**^	−0.3511	−0.0690
LR	62.2000					
R^2^	0.2400					

^**^, ^*^Represent significance levels of 1 and 5%, respectively.

## Discussion

This study applied DEA to comprehensively evaluate the service efficiency of human and non-human resources in LTCFs in different time periods and regions. By combining the spatial Markov chain and spatial correlation, we explored the heterogeneity and correlation of service efficiency of LTCFs in different regions and their interactive effects to provide a reference basis for optimizing the allocation of care resources and improving service efficiency.

### The evolutionary trends of the efficiency of LTCFs from 2013 to 2020

The number of LTCFs, which achieve optimal efficiency in TE, PTE, and SE, presented a composite W-shape changing trend from 2013 to 2020. During 2013–2015, the number of LTCFs with optimized service efficiency showed a downward trend in volatility. After reaching the peak in 2016, the number gradually decreased, reached the “trough” in 2019, and showed an upward trend in 2020. Taking 2016 as a reference, the front and back fluctuations show a symmetrical distribution, that is, the quantitative changes in the optimization of service efficiency of LTCFs form a circular rotation phenomenon in different years. The number of LTCFs with optimized efficiency reached a peak in 2016 and 2020, respectively, which might be due to the policy support of the three-year action plan for eldercare services (2014–2016) issued by the Ministry of Civil Affairs (75). In 2017, the State Council issued the “13th Five-Year Plan for the Development of the National Cause for Aging and the Construction of the Eldercare System”, which highlighted that the eldercare service system based on home, supported by communities, supplemented by institutions, and combined with medical care will be more complete (76). Subsequently, the policy and economic supports mainly focus on the development of home-based community eldercare services. This has led to a downward trend in the service efficiency of LTCFs since 2017. Specific phenomena include the decline in bed utilization rate and low service quality in LTCFs (77). However, the Ministry of Civil Affairs' Implementation Opinions on Further Expanding the Supply and Promoting the consumption of eldercare services in 2019 proposed that LTCFs and community eldercare service institutions should jointly provide support for home care (78). This further expands the market service demand of LTCFs and improves the efficiency of institutions. Taking 2020 as an example, the overall efficiency of institutions in all provinces in China is evaluated. The results show that the TE of LTCFs in 16 provinces (51.61%) is 1, the SE remains unchanged, and the TE of LTCFs in the other 15 provinces is lower. The reasons for low efficiency include the decline of scale efficiency and pure technical efficiency. SE refers to the impact of industrial structure on output efficiency through continuous optimization and improvement of its own allocation; PTE reflects the internal management and personnel management level of LTCFs. Due to the regional distribution and functional diversity of natural resources in China, provinces with high SE are mainly distributed in Northwestern regions with wide geographical areas and rich land resources (19) such as Henan, Tibet, and Xinjiang (79). Hence, the comprehensive efficiency of LTCFs in Northwestern regions is less likely to be affected by SE. The southeastern regions, such as Jiangsu, Shandong, Guangdong, and Chongqing, have a lower SE due to the characteristics of land supply scarcity. As their PTE is high, the reduction in the comprehensive efficiency of the above regions is mainly due to their low-scale efficiency (80). The Malmquist cross-period (2013–2020) analysis results also show that the decline of total factor productivity of LTCFs in the provinces of Northwestern regions is mainly caused by the decline of technical level (13), which is in accordance with the opinion from Torabipour that technical levels played a major role in total productivity changes (81).

### The dynamic evolution of the efficiency of human resources in LTCFs

The output and input efficiency indicators of LTCFs were divided into human and non-human resources, and the situation of the two periods 2013–2017 and 2018–2020 was analyzed separately. The results showed that the development of service efficiency of LTCFs in China is relatively stable and sustainable in the short-term period. This indicated that the convergence trend of the efficiency of LTCFs is increasing, with a gradual decrease in liquidity as time periods were extended (82). However, the transfer probability changed slowly between adjacent levels, which is similar to the test result of spatial autocorrelation, indicating a lower probability of achieving short-term cross-stage development (83). The regions are difficult to realize the transition by relying on their own development without any external causes. The reason for this might be LTCFs focused on the industrial accumulation in the early stage, forming a high homogeneity, and strong substitutability, with insufficient coordinated development, weakening the inter-provincial spatial connection, and poor liquidity (84, 85). Sufficient human resource embeddedness can not only promote the technical development of the eldercare service industry in the region but also play a greater role in regional diffusion than agglomeration; that is, it has a positive impact on the development of the pension industry in neighboring provinces (86–88). In human resource efficiency, our results showed that there was significant spatial polarization without spatial correlation among regions, making it difficult to develop area linkages. The reason for this might be that the staff have relatively stable regional characteristics, weakening the inter-provincial spatial connection. We found that female workers aged between 35 and 45 years can positively affect the efficiency of LTCFs. The efficiency growth is highly dependent on those workers with proficient skills (89). Those staff, at this age range, can stay with high workloads in order to refine and improve their existing skills in performing tasks efficiently, and in the process, complete their work with passion. They are expected to have more patience and dedication to eldercare so that they can obtain better service quality and then improve the efficiency of LTCFs in the process (30). However, younger staff aged < 35 years do not have any sense of longevity with their jobs due to their lack of sufficient skills or have more determinants of intention to leave jobs than older staff in LTCFs (90). Moreover, older staff aged more than 45 years stay focused and are less likely to improve their existing skills. They perform highly repetitive care tasks in a timely manner and complete their assignments within a tight work schedule (91). With restricted personal resources, they can only work in a highly regulated environment and tend to be job burnout and exhausted (90). So, increasing female workers aged between 35 and 45 years can enhance the growth of service quality and personnel stability of LTCFs, in turn, improving the service quality and the overall efficiency of LTCFs. In addition, the type of service subjects has a positive impact on the efficiency of LTCFs. A possible reason for this is that those groups are generally in poorer health or able to receive more government support and subsidies from long-term care insurance, so this income increases the efficiency of LTCFs. Therefore, strengthening policy support to encourage inter-regional initiatives, particularly highlighting the development of human resources interaction and common development, is urgent.

### The dynamic evolution of the efficiency of non-human resources in LTCFs

Non-human resources like construction space can also affect the temporal and spatial changes of the efficiency in LTCFs (92). More specifically, non-human resource efficiency presented a “cluster” distribution mode, demonstrating a certain linkage among various provinces, indicating a great probability to achieve cross-stage development, particularly in the later period. Economic development and land resource gaps might be the main reasons for regional disparities. Non-human resources, like economic development level, environmental regulation, education level, and resource endowment indicators, are all important prerequisites and guarantees for the development of the ability of the eldercare industry (93), but they have little impact on the development of technological innovation ability in surrounding provinces and do not have an obvious spatial spillover effect. Tobit regression analysis results also showed that the comprehensive efficiency of LTCFs decreases by 0.210 for every square increase in construction space variation. The appropriate scale for technical support is an important factor in improving the quality of the facilities (33). Excessive scales will increase care service consumption and cause problems such as deficits. Lower scales will affect comprehensive efficiency and reduce the ability of LTCFs to attract older adults in need of care and improve care technology and quality. Our results demonstrate that there is an urgent trade-off between resource reduction, construction excessive, and substantial regional variation in efficiency. Therefore, it is necessary to avoid excessive construction space changes and improve technology and service quality changes to further improve the efficiency of LTCFs.

## Conclusion

This study provided an analysis of the phased characteristics, cross-temporal distribution, regional distribution, spatial correlation characteristics, and the analysis of influencing factors of the innovative development of China's eldercare service industry. The service efficiency of LTCFs in various provinces has a large regional difference and fluctuation range. The economic development and land resources gap might be the main reasons for the regional disparity. The continuous differentiation of the spatial pattern of service efficiency of LTCFs in various provinces is the performance of the stability of the spatial pattern, indicating less probability to achieve short-term cross-stage development as a whole. To be more specific, the non-human resources efficiency presents a “cluster” distribution mode, demonstrating a certain linkage among various provinces, which causes a great probability to achieve cross-stage development, particularly in the later period. Blindly increasing the construction space and scales of LTCFs does not necessarily increase the output effectively. However, with regard to human resource efficiency, there is a significant spatial polarization without spatial correlation among regions, which makes it a failure to develop by area linkage. The age and gender distribution of staff might increase the inter-provincial spatial differences. As for the supply of resources, the government should command and dispatch the human and non-human resources in the whole country through a top-down design based on the characteristics of each province.

The government must formulate relevant policies to cultivate eldercare staff, optimize the allocation of resources, and enhance quality supervision to increase the service efficiency of LTCFs as a whole. There is also a need to take measures to deal with the spatial polarization limitations of human resources in LTCFs. For example, policymakers should encourage inter-regional drives and the interaction of human resources to promote the common development of the eldercare industry in various regions. Managers of LTCFs should pay more attention to demand-oriented, combined with the policy, and strengthen the institutional operation and management, thus achieving the common development of service efficiency and quality.

### Strengths and limitations

According to the Pareto optimal theory, we selected the input-output indicators for the efficiency evaluation of LTCFs from the aspects of human and non-human resources to get a more refined basis for the development of LTCFs. The spatial Markov matrix was used to conduct continuous dynamic research on the efficiency of the services by considering areas and time heterogeneity. To make a scientific and accurate conclusion, we analyze the local spatial autocorrelation aggregation analysis of the service efficiency of LTCFs between adjacent provinces and their transfer condition. In a long-term period, we could find out the continuous and real changes in human resources and non-human resources efficiency of LTCFs in the development process, and explore the influencing factors leading to temporal and spatial changes.

In this study, the longitudinal data was used to analyze the temporal and spatial pattern of service efficiency in China's LTCFs, and further comprehensive and municipal data still need to be included in the future. More measurement design should be considered to further explore the potential influencing mechanisms of spatial differences in LTCFs. The efficiency changes of both human and non-human resources calculated in this study are compared, but they cannot represent the changes in all the efficiency characteristics of LTCFs. The index data of eldercare service efficiency classification must be mined from multiple dimensions.

## Data availability statement

The original contributions presented in the study are included in the article/Supplementary material, further inquiries can be directed to the corresponding author.

## Author contributions

YH, LZ, and YF worked together. YH was in charge of the study design. LZ analyzed and interpreted the data and drafted the manuscript. YF and YH participated in the statistical analysis and manuscript drafting. YF and LZ supervised and revised the manuscript. All authors have revised, read, and approved the final manuscript.
