# Supplementary material for: Non-human and human service efficiency of long-term care facilities in China
Source: Front Public Health. 2023 Mar 2;11:1066190. doi: 10.3389/fpubh.2023.1066190 (PMC10018177; doi:10.3389/fpubh.2023.1066190)
Supplement: Supplementary file 1 [file Data_Sheet_1.docx]

**Supplementary Table S1** Input-output index system of human resources and non-human resources efficiency

| Indicator type | Dimensions | Indicators |
| --- | --- | --- |
| Input | Human resources | The number of social workers |
|  |  | The number of employees |
|  | Non-human resources | The number of institutions |
|  |  | The original price of fixed assets |
|  |  | The number of beds at the end of the year |
| Output | Service efficiency | Operating income |
|  |  | The number of independents |
|  |  | The number of partially disabled |
|  |  | The number of disabled |
|  |  | The number of rehabilitation and medical outpatients |

**Supplementary Table S2.** Summary statistics of input-output variables (from 2012 to 2016)

|  | Outputs | | | | |  | Inputs | | | | |
| --- | --- | --- | --- | --- | --- | --- | --- | --- | --- | --- | --- |
|  | O1 | O2 | O3 | O4 | O5 |  | I1 | I2 | I3 | I4 | I5 |
| 2012 |  |  |  |  |  |  |  |  |  |  |  |
| Mean | 152148 | 65453 | 14604 | 7221 | 218607 |  | 1429 | 10671 | 239816 | 63 | 127989 |
| Min | 12908 | 2049 | 418 | 5 | 15 |  | 79 | 513 | 10158 | 1 | 7199 |
| Max | 961553 | 225829 | 44380 | 30246 | 1074797 |  | 3386 | 29491 | 1041701 | 209 | 365270 |
| 2013 |  |  |  |  |  |  |  |  |  |  |  |
| Mean | 169846 | 67221 | 15904 | 7865 | 241950 |  | 1370 | 11481 | 274530 | 75 | 138552 |
| Min | 3774 | 3389 | 392 | 258 | 105 |  | 78 | 601 | 26545 | 4 | 7935 |
| Max | 497703 | 232059 | 48926 | 28246 | 1720412 |  | 3336 | 30914 | 858955 | 223 | 399449 |
| 2014 |  |  |  |  |  |  |  |  |  |  |  |
| Mean | 142765 | 55502 | 14800 | 7606 | 208109 |  | 2978 | 15359 | 290807 | 96 | 180558 |
| Min | 3696 | 2574 | 440 | 105 | 350 |  | 86 | 627 | 20523 | 3 | 6943 |
| Max | 470109 | 240348 | 52511 | 29134 | 955228 |  | 22305 | 54869 | 1193418 | 290 | 554479 |
| 2015 |  |  |  |  |  |  |  |  |  |  |  |
| Mean | 138312 | 44347 | 12948 | 7600 | 269197 |  | 895 | 10266 | 234295 | 100 | 115528 |
| Min | 2176 | 1181 | 217 | 16 | 3605 |  | 35 | 381 | 8897 | 3 | 5022 |
| Max | 492278 | 180009 | 46398 | 29471 | 1436994 |  | 2547 | 30717 | 851817 | 353 | 388561 |
| 2016 |  |  |  |  |  |  |  |  |  |  |  |
| Mean | 157470 | 44035 | 14124 | 8463 | 244204 |  | 922 | 10929 | 260846 | 132 | 122186 |
| Min | 11630 | 815 | 22 | 29 | 3535 |  | 7 | 91 | 22070 | 4 | 1124 |
| Max | 524550 | 174632 | 48365 | 32577 | 1187467 |  | 2573 | 33463 | 1008513 | 664 | 410441 |

*Note.* O1,operating income; O2, the number of independents; O3, the number of partially disabled; O4, the number of the disabled;O5, the number of rehabilitation and medical outpatients; I1the number of institutions; I2, the number of employees at the end of the year; I3, the original price of fixed assets; I4, the number of social workers;I5 the number of beds at the end of the year.

**Supplementary Table S3.** Correlation level between input indicators and output indexes

| Out-input indicators | Operating income | The number of independents | The number of partially disabled | The number of disabled | The number of rehabilitation and medical outpatients |
| --- | --- | --- | --- | --- | --- |
| The number of institutions | 0.532^**^ | 0.950^**^ | 0.912^**^ | 0.681^**^ | 0.543^**^ |
| The number of employees | 0.778^**^ | 0.777^**^ | 0.913^**^ | 0.924^**^ | 0.701^**^ |
| The original price of fixed assets | 0.823^**^ | 0.812^**^ | 0.825^**^ | 0.727^**^ | 0.721^**^ |
| The number of social workers | 0.835^**^ | 0.560^**^ | 0.691^**^ | 0.794^**^ | 0.715^**^ |
| The number of beds at the end of the year | 0.688^**^ | 0.906^**^ | 0.938^**^ | 0.801^**^ | 0.621^**^ |
| ** indicates a significant correlation level of 0.01(double-tailed) | | | | | |

**Supplementary Table S4.** Analysis on the reasons for the low overall efficiency of LTCFs in 15 provinces in 2020

| No. | Provinces | Cause analysis of efficiency change |
| --- | --- | --- |
| 3 | Hebei | Pure technical efficiency and scale efficiency are not high, and the return to scale remains unchanged |
| 4 | Shanxi | Pure technical efficiency and scale efficiency are not high, and the return to scale remains unchanged |
| 5 | Inner Mongolia | Pure technical efficiency and scale efficiency are not high, and the return to scale remains unchanged |
| 8 | Heilongjiang | Pure technical efficiency and scale efficiency are not high, and returns to scale are decreasing |
| 10 | Jiangsu | Scale efficiency decreases and returns to scale decline |
| 12 | Anhui | Pure technical efficiency and scale efficiency are not high, and the return to scale remains unchanged |
| 13 | Fujian | Pure technical efficiency and scale efficiency are not high, and the return to scale remains unchanged |
| 15 | Shandong | Scale efficiency decreases and returns to scale decline |
| 19 | Guangdong | Scale efficiency decreases and returns to scale decline |
| 20 | Guangxi | Pure technical efficiency and scale efficiency are not high, and the return to scale remains unchanged |
| 22 | Chongqing | Scale efficiency decreases and returns to scale decline |
| 24 | Guizhou | Pure technical efficiency and scale efficiency are not high, and the return to scale remains unchanged |
| 25 | Yunnan | Pure technical efficiency and scale efficiency are not high, and the return to scale remains unchanged |
| 27 | Shaanxi | Scale efficiency decreases and returns to scale decline |
| 28 | Gansu | Pure technical efficiency and scale efficiency are not high, and the return to scale remains unchanged |
|  | | |

**Supplementary Table S5.** Time homogeneity test (2013-2017, 2018-2020)

| Testing statistics | df | *P* value |
| --- | --- | --- |
| LR=33.733 | 6 | 0.005 |
| Q=96.23 | 6 | <0.001 |


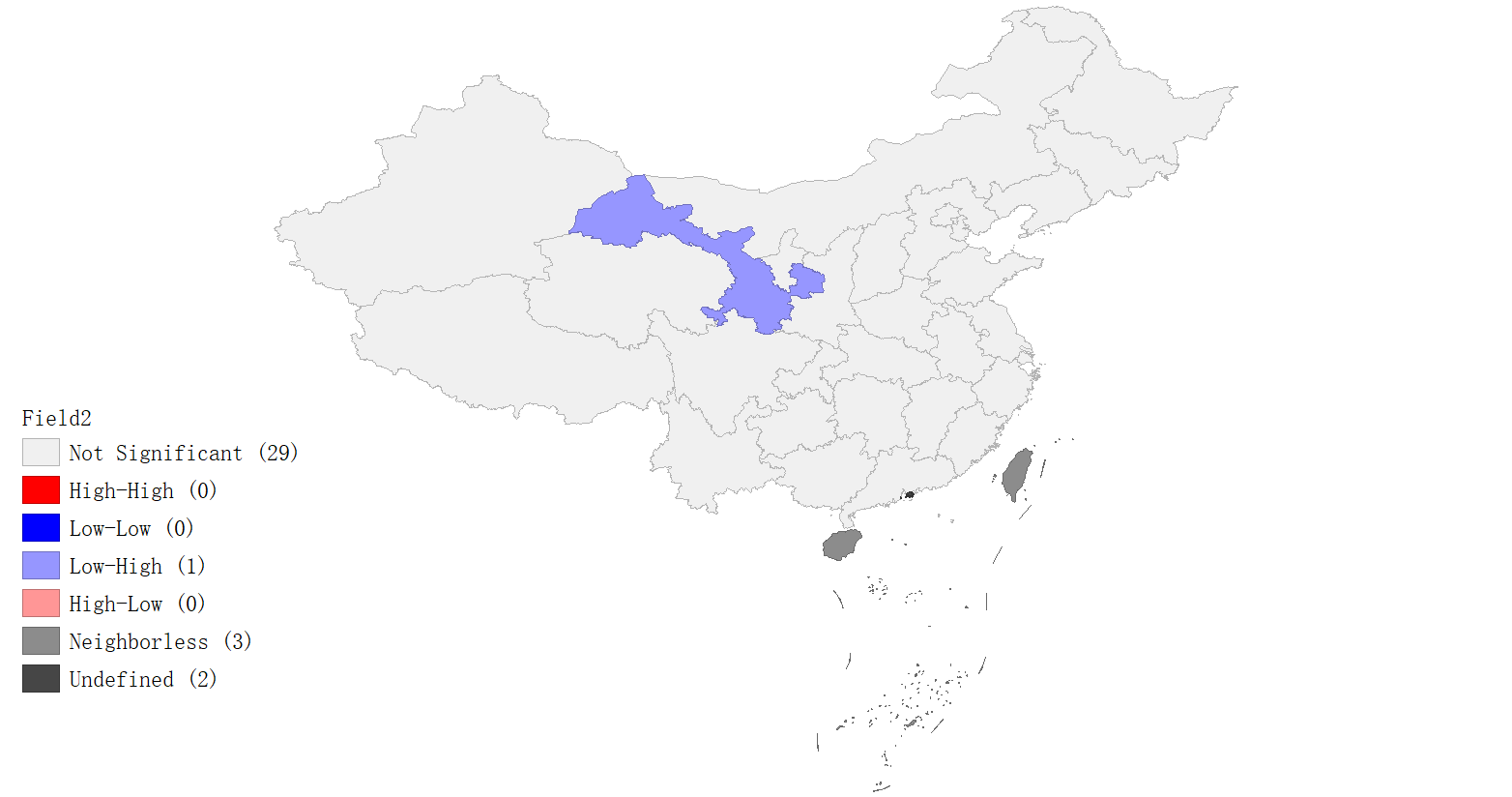


2013 Year (a)


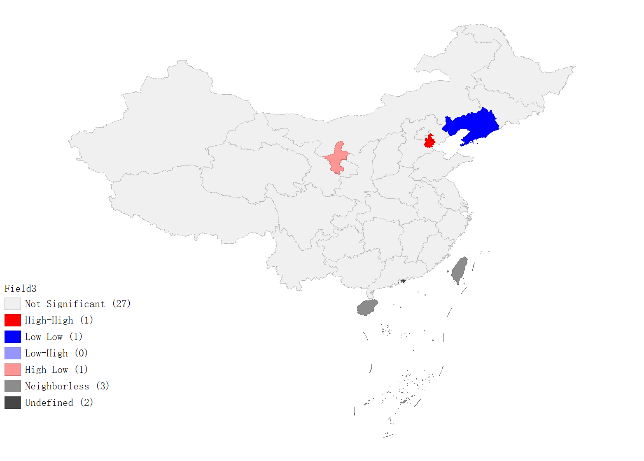


2014 Year(b)


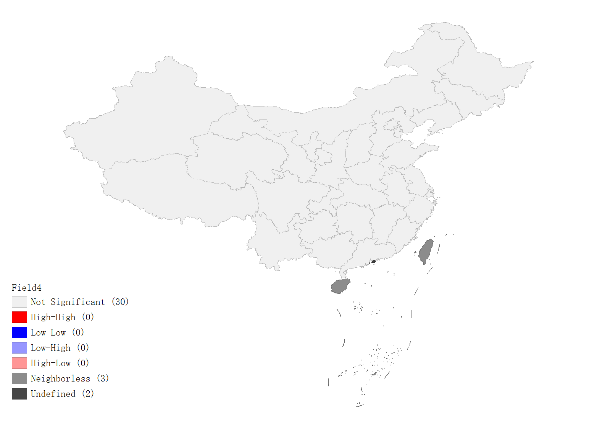


2015 Year(c)


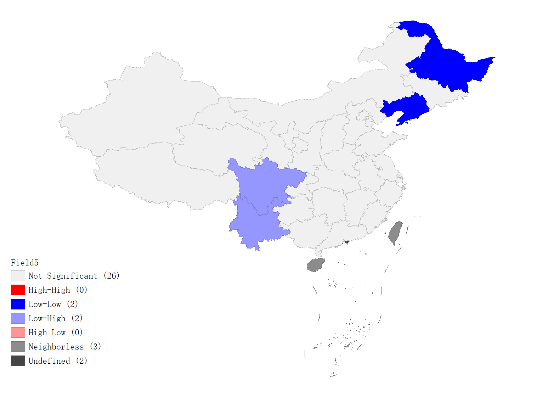


2016 Year(d)


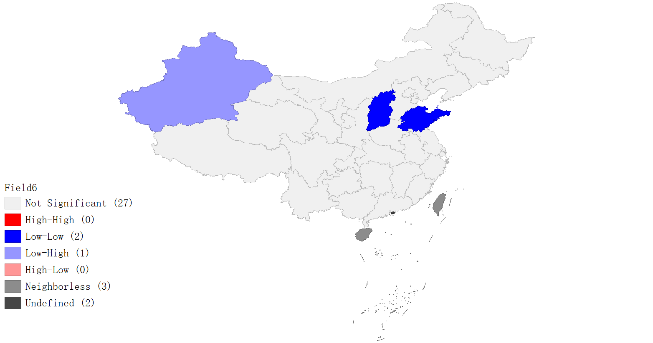


2017 Year(e)


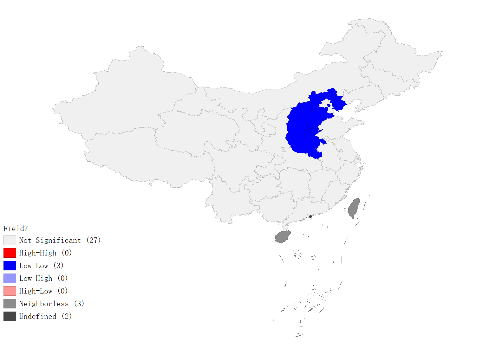


2018 Year(f)


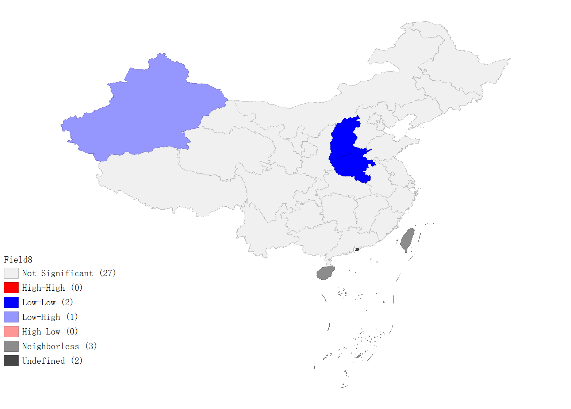


2019 Year(g)


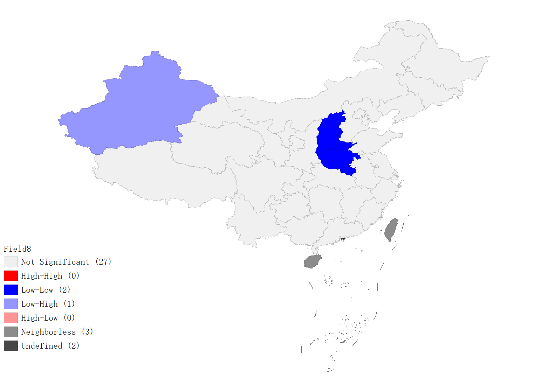


2020 Year(h)

**Supplementary Fig. S1a-h** Local spatial autocorrelation aggregation diagram of service efficiency of elderly care institutions in various provinces from 2013 to 2020
